# Oxoisoaporphines and Aporphines: Versatile Molecules with Anticancer Effects

**DOI:** 10.3390/molecules25010108

**Published:** 2019-12-27

**Authors:** Esteban Rodríguez-Arce, Patricio Cancino, Manuel Arias-Calderón, Paul Silva-Matus, Marianela Saldías

**Affiliations:** 1 Instituto de Investigación e Innovación en Salud, Facultad de Ciencias de la Salud, Universidad Central de Chile, Santiago 8370178, Chile; esteban.rodriguez.arce@gmail.com; 2 Facultad de Ciencias Químicas y Farmacéuticas, Universidad de Chile, Santiago 8380544, Chile; pcancino@ciq.uchile.cl; 3 Departamento de Ciencias Biológicas, Facultad de Ciencias de la Vida, Universidad Andres Bello, Santiago 8370146, Chile; manuel.arias@unab.cl; 4 Departamento de Ciencias de la Salud, Universidad de Aysén, Coyhaique 5951537, Chile; paul.silva@uaysen.cl

**Keywords:** telomerase inhibition, alkaloids, oxoisoaporphine, aporphine, boldine, ROS, anticancer action, sampangine, structure–biological activity

## Abstract

Cancer is a disease that involves impaired genome stability with a high mortality index globally. Since its discovery, many have searched for effective treatment, assessing different molecules for their anticancer activity. One of the most studied sources for anticancer therapy is natural compounds and their derivates, like alkaloids, which are organic molecules containing nitrogen atoms in their structure. Among them, oxoisoaporphine and sampangine compounds are receiving increased attention due to their potential anticancer effects. Boldine has also been tested as an anticancer molecule. Boldine is the primary alkaloid extract from boldo, an endemic tree in Chile. These compounds and their derivatives have unique structural properties that potentially have an anticancer mechanism. Different studies showed that this molecule can target cancer cells through several mechanisms, including reactive oxygen species generation, DNA binding, and telomerase enzyme inhibition. In this review, we summarize the state-of-art research related to oxoisoaporphine, sampangine, and boldine, with emphasis on their structural characteristics and the relationship between structure, activity, methods of extraction or synthesis, and anticancer mechanism. With an effective cancer therapy still lacking, these three compounds are good candidates for new anticancer research.

## 1. Introduction

Since its discovery, cancer remains one of the most difficult diseases to treat, emerging as the second cause of death globally according to World Health Organization statistics. In 2018, cancer and its associated pathologies were responsible for nearly 10 million deaths globally [1]. Effective cancer treatment is lacking due to the mechanisms and characteristics of cancer, including the biological and genomic complexity of this disease, which place continual pressure on researchers to develop new therapies for cancer treatment.

Since the discovery of the anticancer properties of nitrogen mustard family agents [2], identified for initial chemotherapy cancer treatment, the anticancer properties of natural organic compounds have been increasingly studied. The analysis of the chemistry of natural compounds to search for anticancer properties has included the characterization of the cytotoxic effect of different natural extracts and their derivates, to the rational design and synthesis of molecules to target some of the molecular actors in cancer signaling, including proteins, enzymes, receptors and/or nucleic acids, and the compounds have been tested in several cancer models, both in vitro and in vivo [3,4]. 

The use of plant extracts as cancer treatment started in the ancient civilizations and continued until present as one of the chemical bases for anticancer therapies. Since the discovery of the cytotoxic properties of vinblastine and vincristine, two alkaloids extracted from *Catharanthus roceus*, the National Cancer Institute has supported large-scale screening programs for antitumor agents, leading to the report of over 50,000 natural compounds with antitumoral properties. More than 70% of the Food and Drug Administration (FDA)-approved molecules are organic compounds from natural extracts or modified organic structures from a natural compound, including sampangines and oxoisoaporphine family molecules [5,6]. Boldine is 1 of 18 alkaloids has been detected in the bark, leaves, wood, and roots of *Peumus boldus*, standing out due to its hepatoprotective, cytoprotective, anti-inflammatory, and choleretic properties, and its characteristic alkaloids with a high polyphenols content. Boldine was reported to prevent both enzymatic- and non-enzymatic-mediated damage to biological systems [7]. 

To understand which mechanism in cancer biology is targeted by an organic compound is crucial for developing a modified organic structure with better antitumoral and pharmacology parameters. However, several biological mechanisms could be targeted by a drug with anticancer properties. Several mechanisms have been described as anticancer targets, including inhibition of nucleic acid synthesis and/or DNA replication [8,9], reactive oxygen species (ROS) generation [10,11], cell cycle arrest [12,13], apoptosis [14], and cytoskeleton inhibition [15].

For sampangines and oxoisoaporphine and their derivatives, the molecular mechanisms involved in the anticancer activity of these compounds are mainly related to DNA interaction forming G quadruplex (G4) complexes, inhibition of the telomerase activity, and ROS generation. These mechanisms all lead to cancer cell death.

Telomere and telomerase activity are both important targets in anticancer therapy, mostly by design, and new molecules are capable of interacting with these structures to inhibit these physiological functions. Telomeres are repetitive nucleotide sequences located at the end of chromosomes that terminate in a single-stranded TTAGGGn sequence associated with a multiprotein complex called the shelterin complex, which forms a shield to protect the telomeric end of each chromosome from DNA damaged, providing a repair mechanism in mammalian cells [16,17]. Shelterin complex not only protect chromosome endings, also regulates telomerase function, controlling the access of the telomerase for telomeric binding. This function associated with control of the recruitment of telomerase has been pointed as a new anticancer target [18]. Telomeres progressively shorten during each round of cell replication due to incomplete lagging-strand DNA replication, oxidative damage, and other factors. The shortening of telomeres is key biomarker of the senescence process of cells and eventually generates cell cycle arrest and cell death via apoptosis [19]. The nucleotide sequence is elongated via telomerase enzyme activity a ribonucleoprotein complex whose function is to synthesize and to elongate telomeres, which prevents the shortening of these regions and cell senescence [20]. So, telomerase activity confers immortality and limitless proliferation of cells that express this enzyme, which is normally repressed in healthy mammalian cells. The understanding this mechanism leads to the investigation of the participation of telomerase in cancer development. About 80%–90% of human cancers constitutively express telomerase, indicating the importance of the inhibition of this enzymatic activity in anticancer therapy [21]. Different natural and synthetic compounds have been proposed as telomerase inhibitors in anticancer therapy, including oxoisoaporphine-related molecules, based on their capacity to inhibit telomerase components [22]. Basically, telomerase is constitute by a catalytic subunit with reverse transcriptase activity known in humans as hTERT, and by a ribonucleotide template (hTR), which is essential for the elongation of telomeric repetitive nucleotide sequences [23]. The inhibition of telomerase is mostly based in the down-regulation of hTERT expression, direct competition for hTERT interaction, or blockade of hTERT nuclear translocation [22,24]. The oxoisoaporphine molecules and their derivatives present an anticancer mechanism based on their binding to different structural and functional domains of telomerase, described by docking experiments [25,26]. In the oxoisoaporphine section of this review, a detailed description of the structure of these molecules and the interaction with telomerase is provided.

Some natural compounds exert their anticancer activity by stabilizing a non-canonical DNA secondary structure, known as G-quadruplex (G4). G4 structures are formed by stacked guanine tetrads (also known as G-tetrads), within four guanine bases arranged in a square plane conformation, stabilized by Hoogsteen hydrogen bonding, instead of the classical Watson–Crick nucleotide interaction [27]. G4 DNA is located predominantly in telomeric sequences and the promoter regions of several oncogenes, like *c-Myc* and *c-kit* [28,29]. The stabilization of the G4 structures, in both telomeric or promoter regions, inhibit the specific enzymatic activity in that DNA zone. The formation of G4 at a telomeric region blocks the telomerase hybridization and catalytic elongation of the telomere, thereby altering the telomere, which produces a process that occurs in cancer cells, reducing proliferation and leading to cell death [30]. G4 formation at oncogene promoter regions must be unwound to achieve proper transcription of the gene. So, the stabilization of this G4 promoter structures blocks the transcription process, leading to a down-regulation of oncogenes expression. For these reasons, the use of molecules that bind and stabilize the G4 structure is now a research focus for anticancer therapy, resulting in several natural and synthetic molecules characterized as G4-ligands [31].

For sampangine and derivates of this family, their anticancer mechanism is not achieved by a direct interaction with DNA or telomerase, but is mainly through ROS production [32]. ROS are highly reactive molecules that are partially reduced oxygen derivates, which have a single unpaired electron acting as a second messenger in different cellular processes, both in normal and cancer cells [33]. The ROS molecules include superoxide (O_2_^•−^), hydroxyl radical (•OH), nitric oxide (NO•), hydrogen peroxide (H_2_O_2_), and singlet oxygen (^1^O_2_), among others, with hydrogen peroxide, superoxide, and hydroxyl radicals being the most studied ROS in cancer [34]. Cellular sources of ROS include the metabolic process in mitochondria [35] and the NADPH oxidase complex enzyme activity [36]. ROS levels are elevated in cancer cells compared with normal cells due to an increase metabolic rate, oncogene activity, and mitochondrial dysfunction [37], and related to a diminished antioxidant enzyme activity [38]. The elevated oxidative stress status in cancer cell plays an important role in the development of cancer as it regulates both genomic and non-genomic effects that induce cancer in humans [10]. However, excessive ROS production can have anticancer effects, mostly by triggering cancer cell death by apoptosis [39] and up-activation of tumor-suppressor genes forcing irreversible cellular senescence [40]. Based on these reports, the natural molecules that induce changes in ROS level in cancer cells can potentially be used in anticancer therapy. ROS-related chemotherapy and the role of ROS in cancer have been thoroughly reviewed [11,33,34,41].

Hanahan and Weinberg proposed that the rationale of anticancer therapy-design should be based on the ability of the intervention to interfere with at least one of the hallmarks of cancer [42].

Hallmarks of cancer are well-defined featuring involved in cancer development and progression, including resistance to cell death, metabolism reprogramming, enably replicative immortality and genome instability and mutation, among others [42,43].

Anticancer mechanisms should diminish or abolish at least one of these hallmarks for an efficient anticancer effect. Oxoisoporphines and sampangines both impact on hallmarks through their anticancer effects, which reinforced the idea that these compounds could be promising anticancer agents for therapy designs. 

In the case of oxoisoporphines and their derivates, the anticancer effects described for this molecule interferes with the replicative immortality capacity and cell death avoiding features of cancer cells, through the interaction with DNA telomeric sequences and inhibition of telomerase activity, and apoptosis induction respectively [44].

In the case of sampangines compounds, their mechanisms of action are related with induced cell death via apoptosis through ROS modulation [31,32] interfering with immortality and avoiding cell death hallmarks of cancer cells. Nevertheless, more research could be developed to fully understand the effects of oxoisoporphines and aporphines with hallmarks of cancer cell, as a part of the rationale design of cancer therapies based on this molecule.

## 2. Anticancer Natural Compounds: Oxoisoaporphines, Sampangines, and Boldine—Synthesis and Structural Description

### 2.1. Oxoisoaporfines

Oxoisoaporphine alkaloids (7*H*-dibenzo [de,h]quinolin-7-one) are a family of organic molecules used in the treatment of multiples diseases [45,46,47,48]. The biological effects of this family of molecules has been observed in the treatment against several types of cancer [48] and Alzheimer’s disease [49], and in anti-depressant and anti-leishmanial activities in mice [50,51]. It is isolated from the rhizome of *Menispermum dauricum* DC, and this plant is very common in China. *M. dauricum* DC has been used in traditional medicine as an analgesic and antipyretic for the treatment of sore throats, colitis, dysentery, and rheumatic arthralgia, supporting its inclusion in the China Pharmacopeia [52,53,54,55,56,57,58,59]. Other properties of this plant have been studied for many groups in the last 40 years, such as antitumor activity [60], as an antiarrhythmic drug [61], and dopaminergic activity in the D1 and D2 receptors in the central nervous system [62]. 

**Figure 1:**
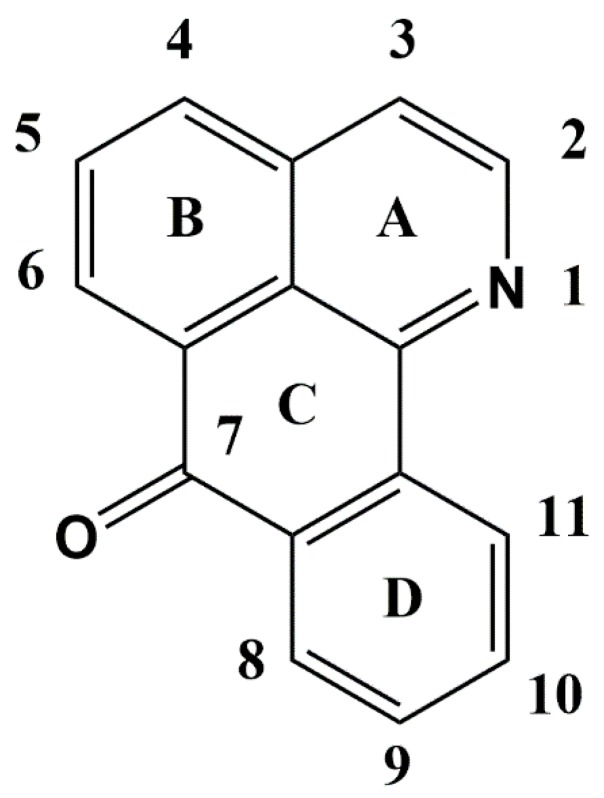
Molecular structure of 1-azabenzanthrone.

The oxoisoaporphine alkaloids are derivatives of 1-azabenzanthrone (Figure 1). They are composed of a quinoline ring attached to a tetralone unit, and both these fragments form a highly aromatic compound that has been completely characterized using different techniques [57,59,63,64]. The nitrogen atom in the skeleton has the same effect in the structure as on electronic density distributions (together with the carbonyl group) in the entire system [54]. This molecule is versatile from a synthetic viewpoint due to the many positions available for incorporating different chemical groups (donor or acceptor) with the aromatic portions (B and D rings). 

The molecule has a planar structure and disposes of the conjugated ring that confers electronic and structural properties that could be potentially used for medicinal applications as an antiparasitic (malaria), anti-Alzheimer’s disease (AD), or anti-tumoral agent [45,46,47]. For example, the 1-azabenzanthrone moiety of oxoisoaporphine alkaloids can interact with DNA via intercalation due to the planar structure; they can be intercalated through the DNA strands, which is the principal mechanism of action responsible for the cytotoxicity of oxoisoaporphine alkaloids [44].

#### Synthesis

Many studies have reported the synthesis of oxoisoaporphine derivatives [46,56,57,63,65,66,67,68]. Most synthesis methods follow a similar process starting with 3-(β-dialkoxyarylethylamino)phthalides with polyphosphoric acid [66,69], β-phenylethylamines with phthalic anhydride, or substituted β-phenylethylamines with benzoyl chloride [44,45,67,68]. The reaction is a Bischler–Napieralski reaction or a variant [70].

**Scheme 1:**
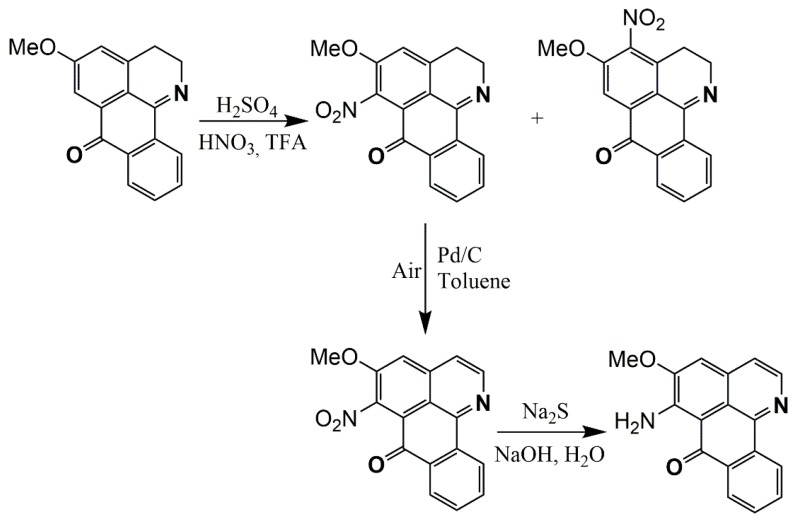
Synthesis of lakshminine (compound **1**) reported by Castro-Castillo et al. [66].

The aim of the synthesis of these compounds is to modify some position in the skeleton of 1-azabenzathrone to increase the anti-cancer activity of the molecules via the incorporation of groups to change the electronic properties as donor or acceptor fragments. The synthesis of these compounds is not challenging, and some research groups have identified efficient synthetic routes [46,56]. For example, Castro-Castillo et al. described a synthesis route from 1-Aza-2,3-dihydro-5-methoxybenzanthrone following these steps: (1) nitration, (2) catalytic oxidation using air, and (3) selective reduction of the nitro groups to obtain lakshminine, as shown in Scheme 1. 

**Scheme 2:**
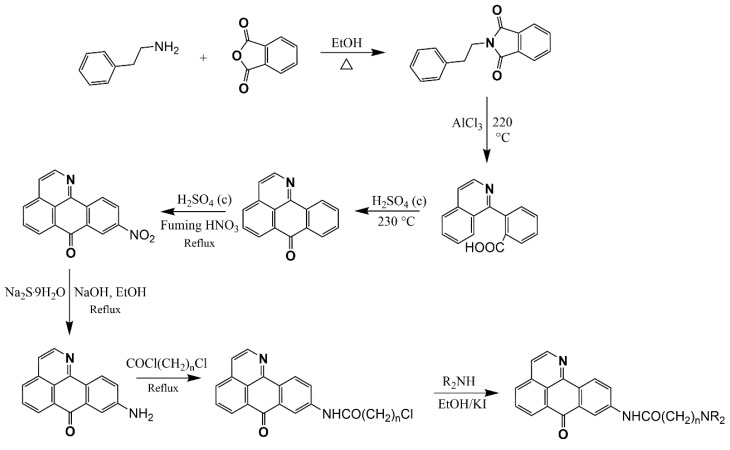
Synthesis route for oxoisoaporphine alkaloid derivates (9-aminoalkanamido-1-azabenzanthrone) [44].

Tang et al. [44] proposed a different synthesis route with more steps, starting with the acylation of β-phenylethylamine with phthalic anhydride to produce phthalimide derivate **1** with 80% yield. Then, two consecutive cyclizations proceed, followed by the nitration of C9. In the final steps, the substituent modified by different reactions over this position is depicted in Scheme 2.

Many approaches to the synthesis of the oxoaporphine ring system have been described by different research groups, typically involving the synthesis of 1-benzyl/1-benzoylisoquinoline intermediates, followed by cyclisation under intramolecular biaryl synthesis, using either photochemical [71,72,73,74,75], radical [76], or Pd-catalyzed [77,78] reactions. 

**Scheme 3:**
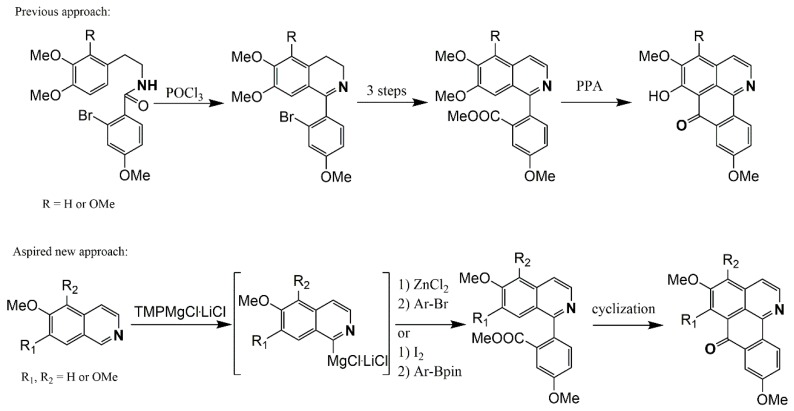
Different routes to obtain oxoisoaporphine alkaloids proposed by Meltzer et al. [83].

Kunitomo described a route for the total syntheses of menisporphine [57] and dauriporphine [56] that starts with the synthesis of 1-(2-bromoaryl) isoquinolines using Bischler–Napieralski chemistry [70], followed by tedious replacement of the bromine substituent by cyanide (for modern variants, see [79,80]), and subsequent conversion to a carboxylate and Friedel–Crafts-type cyclization using polyphosphoric acid (Scheme 3). Along these lines, Meltzer et al. proposed two different methods to obtain menisporphine derivatives. The first is based on synthetic description of Kunitomo [56,57] and the second is a direct metalation of isoquinoline [81], 6,7-dimethoxyisoquinoline [82], and on previously published results of the metalation of various alkoxyisoquinolines [75] at C1 with the Knochel–Hauser base TMPMgCl·LiCl. Transmetalation of the intermediate organomagnesium species with ZnCl_2_ should lead to C1 zincated isoquinolines, which could undergo Negishi cross-coupling reactions with appropriately substituted methyl 2-bromobenzoates to produce methyl 2-(isoquinolin-1-yl)benzoates (Scheme 3) [83]. 

### 2.2. Aporphine Compounds: Boldine and Their Derivatives, Medicinal Application of Natural Aporphines 

Aporphine alkaloids are one of the largest groups of isoquinolines, including structures such as proaporphines, secoaporphines, oxoaporphines, dehydroaporphines, 7-hydroxyaporphines, and aporphinedimmers [84]. Many of those aporphinoids have important pharmacological properties correlated with their chemical moieties and their possible mode of interaction in several environments, such as those promoted by enzymes, proteins, intercalation, or binging DNA.

Boldo (*Peumus boldus*) is an endemic tree in central Chile that is resistant to drought and strong solar radiation and has the ability to regrow from its roots after burning or felling. Around 18 alkaloids have been detected in the bark, leaves, wood, and roots of *Peumus boldus*. Amongst these alkaloids, boldine stands out due to its hepatoprotective, cytoprotective, anti-inflammatory, and choleretic properties, being its most characteristic alkaloid that has a high polyphenol content. (S)-(+)-boldine is the major alkaloid present in the leaves and bark of the Chilean boldo tree, and has been evaluated due to its antioxidant effect that effectively protects different systems against free-radical-induced lipid peroxidation or enzyme inactivation. This activity presumably underlies the hepatoprotective and cytoprotective effects, and may also be related to its antipyretic and anti-inflammatory activity [85,86]. The antiproliferative response has been evaluated on several cancer cell lines, including human breast cancer cell lines such as MDA-MB-231 MCF7, the HepG2 liver cancer cell line, T24 bladder cancer cell line, and the glioma cell line, at non-toxic concentrations and its potential for telomerase inhibition has been reported [87,88]. Paydar et al. showed that boldine induces apoptosis through the cell cycle at the G2/M phase in animal and human invasive breast cancer cell lines. These studies promote boldine as a candidate for telomerase-targeted cancer therapy [89].

**Figure 2:**
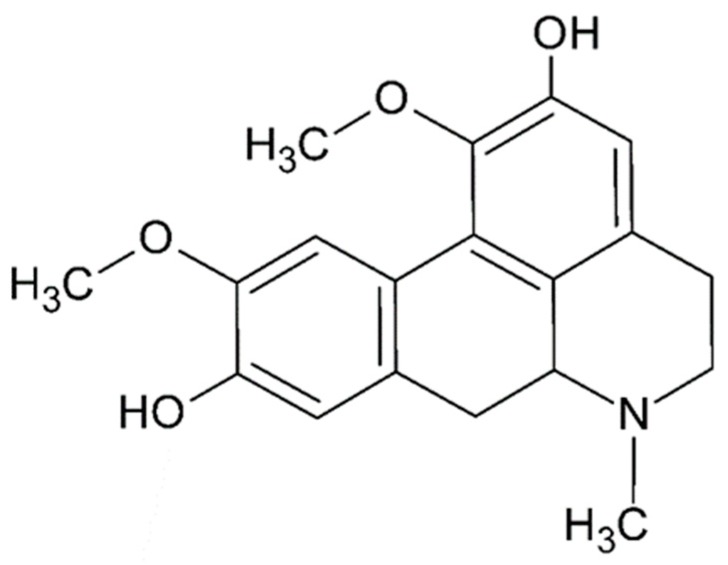
Molecular structure of boldine.

Structurally, (S)-2,9-dihydroxi-1,10-dimethoxyaporphine, known as Boldine (**I**), is formed by four coplanar six-membered rings with two methoxy and two hydroxy groups directly linked to the aromatic rings (Figure 2).

The antioxidant properties of boldine and its derivatives have been evidenced through in vivo and in vitro assays, and attributed to the presence of two phenolic groups attached to a highly conjugated system that also has a benzylamine functionality [90]. The special aporphine conformation in the presence DNA, in some cases, allows for intermolecular interaction between specific DNA sites with protonic substituents from the aporphine moiety. 

#### Extractive Methodology, Synthesis, and Purification

The alkaloid extract of boldo leaves has been studied for decades, initially mixed with other aporphines such as isocorydine, *N*-methyllaurotetanine, and norisocorydine. Later, additional aporphines, including isoboldine, laurotetanine, laurolitsine, and isocorydine *N*-oxide, were identified [91]. Some of these aporphines were later tentatively quantified in boldo leaves and extracts purchased from European suppliers, where boldine was usually found to be a relatively minor alkaloid [92]. The methodology in the European Pharmacopoeia [93] and a modified procedure have allowed the identification of several boldine derivatives in various amounts. Fuentes et al. reported the isolation of alkaloids from bark, wood, roots, and leaves collected near Maria Pinto, Santiago Metropolitan Region, Chile. Using an extractive methodology following the guidelines of the European Pharmacopoeia, they obtained 0.7%, 0.04%, 0.088%, and 0.025% bulk extract and 0.025%, 0.04%, 1.0%, and 0.09% of crude alkaloids in these plant parts, respectively [94]. Of these fractions, the most abundant alkaloid detected was *N*-methyllaurotetanine but not boldine in the total alkaloids. Thus, this derivative was found to be a moderately potent 5-HT_1A_ receptor agonist binding in vitro assays, which might be related to the purported sedative effect of boldo leaf infusions [95]. 

**Figure 3:**
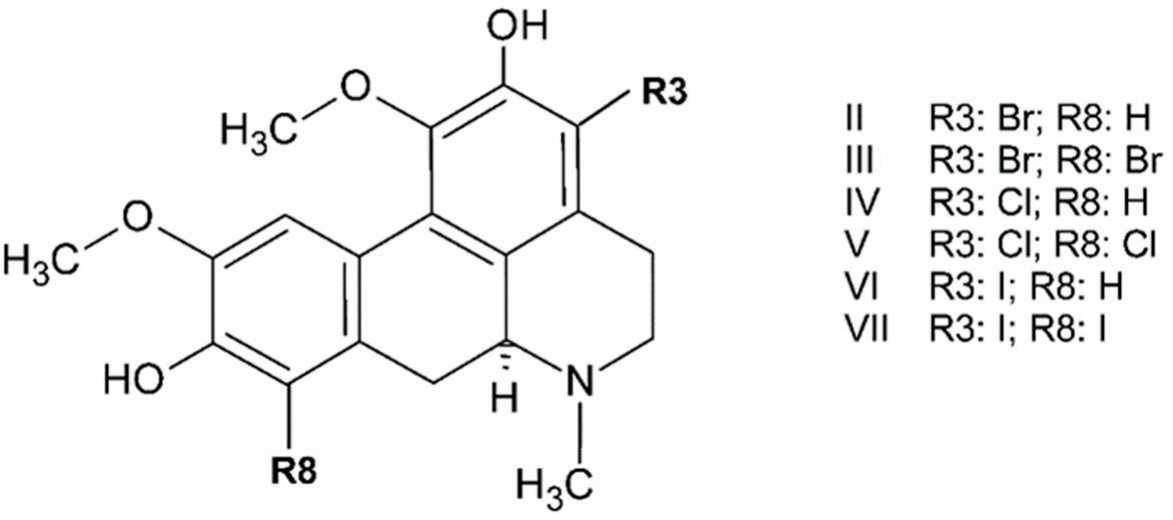
Molecular structure of boldine derivatives determined by Sobarzo et al. [93].

Synthetic boldine derivatives have been related to anti-inflammatory and redox activity. Thus, since the late 1990s, some research on the structure–antioxidative activity of boldine and related compounds suggested that both the phenolic groups attached on the aporphine framework and the basic benzylicamine function contribute to these properties [96]. Some of these derivatives of boldine, from in vivo assays, enhanced its pharmacological properties, and they are expected to be more potent in vivo due to increases of lipophilicty, thereby decreasing intestinal absorption, biotransformation, and excretion. Sobarzo et al. [97] proposed a reasonable functionalization of boldine by incorporating not very bulky substituents, such as chlorine, bromine, and iodine, for increased lipophilicity without considerably affecting the key phenolic and amine functional groups. They reviewed the reaction of boldine with Br_2_ in acetic acid, assuming that protonation of nitrogen might hinder the dehydrogenation side reaction, and they found that a considerable excess of halogen is required. As such, they proved that synthesis using *N*-bromosuccinimide as a bromination agent to ensure the complete protonation of the basic nitrogen atom produces reasonable yields of either 3-bromoboldine (**II**) or the 3,8-dibromo derivative (**III**) (Figure 3). When they studied the chlorination of boldine with *N*-chlorosuccinimide (NCS), 3-chloroboldine at 48% (**IV**; in a 2:1 NCS: boldine ratio) and 3,8-dichloroboldine (**V**) at 19% could also be isolated. The steric and electronic environments of the C3 and C8 of boldine appear to be similar. Consequently, iodination with N-iodosuccinimide (NIS) might be expected to produce both 3-iodoboldine (**VI**) and 3,8-diiodoboldine (**VII**). These results showed that even when using a 2:1 ratio of NIS to boldine, only the 3-iodo derivative could be isolated with a moderate yield.

### 2.3. Sampangine

The sampangines, a class of polycycles copyrine alkaloids, are naturally found in *Cananga odorata*, *Duguetia hadrantha*, and *Anaxagorea dolichocarpa* stem bark. These azaoxoaporphine alkaloids were isolated and their structure elucidated for the first time in 1986 by Rao et al. [98], and their biological activities were reported three years later. Sampangine was isolated as a potential antifungal and antimycobacterial agent, but sampangine also displayed in vitro antimalarial and anticancer activities [99,100,101,102,103]. All these biological properties are related to their basic structure, which corresponds to a class of marine-derived alkaloids named pyridoacridine [102].

**Figure 4:**
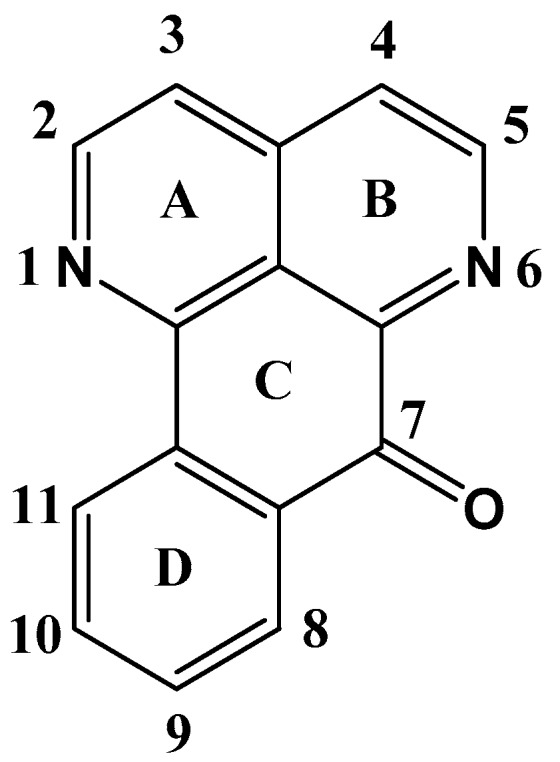
Sampangine molecular structure.

Sampangine is structurally close to the marine alkaloid ascidemin (Figure 4). The structure is composed of four aromatic rings that include two N heteroatoms in the A and B ring, and quinone group (C=O) in the C ring [32,100].

## 3. Oxoisoaporphines, Sampangines, Boldine: Structural and Cytotoxicity Correlation

### 3.1. Oxoisoaporphines as a Versatile Framework for Tuning Bioinorganic Properties

Various routes exist for producing many oxoisoaporphine alkaloids derives. The design of the molecules is important for promoting its potential applications as anti-cancer agents. Most strategies used to design anti-cancer drugs point to the inhibition of telomerase function [104,105,106]. Small molecules, such as oxoisoaporphine alkaloids, can act as interfering ligands, disrupting the telomere–telomerase interaction. The ligand binds the enzyme–substrate to prevent telomere elongation or the formation of the telomere–telomerase complex [107,108]. Therefore, small molecules that selectively bind and stabilize structures, such as telomeric G4 and other G4s DNA, can influence telomere maintenance and potentially serve as therapeutic agents [104,105,106,107,108,109,110,111,112]. Oxoisoaporphine-like analogues were found to have strong DNA binding affinity and therefore high cytotoxicity [44] as well as anti-plasmodial activity [46]. 

**Figure 5:**
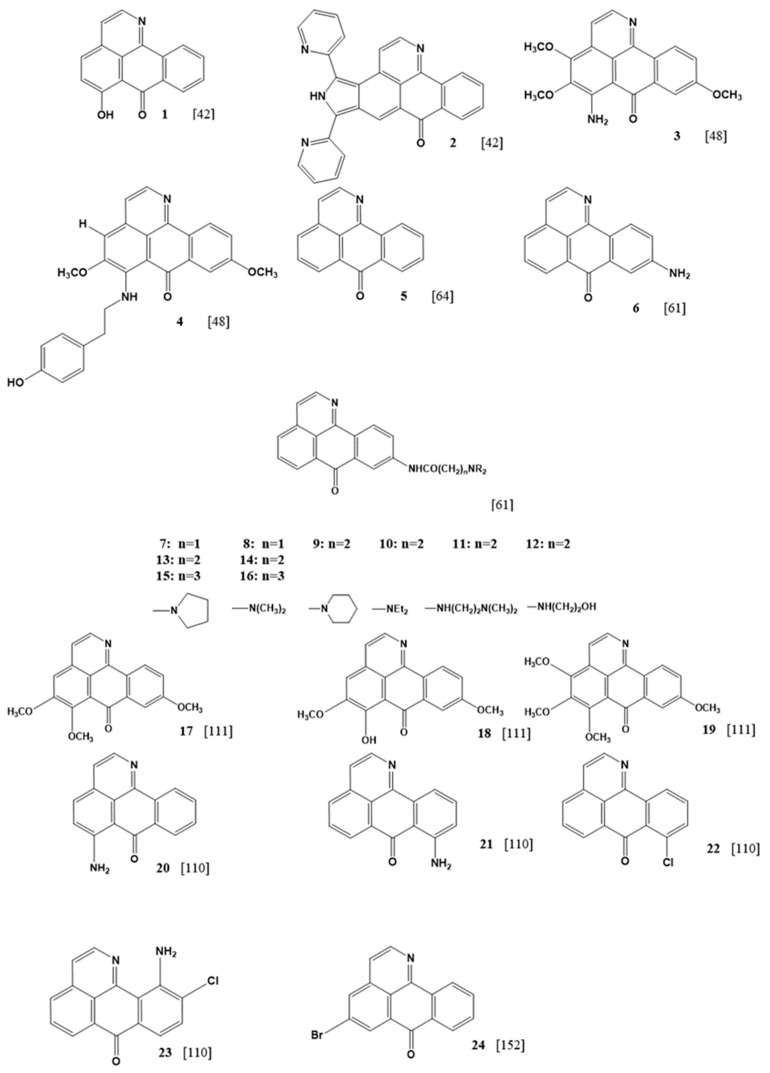
Structure of oxoisoaporphines with cytotoxic assays of IC_50_ against tumoral cell lines.

Figure 5 shows the different oxoisoaporhines on which cytotoxic studies of 50% inhibition (IC_50_) were conducted on a series of tumoral line cells. The results of IC_50_ obtained are summarized in Table 1. A total of 23 compounds that present IC_50_ values in 14 different cells lines were found. Three cells lines are often analyzed due providing more information: hepatocellular carcinoma Hep-G2, human breast cancer MCF-7 and large cell lung carcinoma NCI-H460. The IC_50_ results in Table 1 show that for these cell lines, the concentration ranges between 0.39 and 100 μM. Compounds **9**, **12**, and **14** present higher activity in the two cell lines (Hep-G2 and MCF-7). Gu et al. reported these compounds, and explained that this family of compounds uses DNA binding as their mechanism of action [44]. The results of DNA binding affinity are correlated with cytotoxicity results, and the researchers observed that the compounds with the highest DNA constant binding values had the lowest IC_50_ values. From a chemical point of view, this correlation could be interesting to examine.

Compounds **9**, **12**, and **14** contain only one substitution at the C9 of the 1-azabenzanthrone framework, which is the amide derivative with an amine terminal group (–NHCO(CH_2_)_2_NR_2_), where R varies depending on the compound. The first step is considering that the nitrogen atom from amide bonds directly to the oxoisoaporphine structure, which enhances electro donor groups in the electric cloud of the conjugate system, favoring the interaction of the π-system with the aromatic fragment of the DNA. The R group present in the amine fragment plays an important role in the results. The R group in compound **9** displays a piperidine moiety, **12** shows a secondary amine with a terminal hydroxyl group, and **14** has an alkyl substitution. The best results were found using **12**. Despite the electronic effects, the presence of the hydroxyl group at C9 allows a hydrogen bond that favors DNA binding. 

In contrast, when the substitution at C9 is only amine (compound **6**), the results are moderated, showing that the electronic effect and the ability to generate hydrogen bonds matter, and the longer length of the chain produces better cytotoxic results. Thus, the substitution position has a direct correlation [67] as tested in the same cell lines of compounds only substituted with an amine group in C6 (compound **20**) and C8 (compound **21**) [113]. The results were poor in comparison with the work of Gu et al. as the change in the position of the substituent changed the electronic properties of the compounds. The nearness between the substituents and the ketone reduce the possibility of generating hydrogen bonds, possibly due to an internal hydrogen bond between the oxygen in the carbonyl moiety and the hydrogen in the amine group. This interaction decreases the possibility in both species, reducing the nucleophilic effect of the oxygen of the carbonyl group, and the availability of the hydrogens of the amine group to generate intermolecular interactions, explaining the decrease in the DNA binding affinity of these compounds.

Using the human breast cancer line MCF-7, compounds **9**, **12**, and **14** also maintain high activity (Table 1). However, compounds **3 [52]**, **4 [52]**, and **19 [114]** exhibited excellent results for this cell line. These compounds have substitutions in C4, C5, C6, and C9 that mainly involve methoxy moieties. The substitution at C6 seems to be crucial, which is an amino acid for compound **3**, an aminoalkylphenol for compound **4**, and methoxy group for compound **19**. When comparing the oxoisoaporphine derivatives synthesized by Gu et al. (**9**, **12**, and **14**) with the compounds prepared by Qin et al. (**3**, **4**) and Cheng et al. (**19**) [114], similar results can be observed. However, the best result was obtained for compound **19**, having the lowest IC_50_ among (0.39 µM, Table 1). This derivative presents four methoxy groups attached to the oxoisoaporphine framework. These groups are strong electron donors and increase the electronic density present in the oxoisoaporphine, increasing the electronic density present in the oxoisoaporphine, and allowing better π-π interactions and improve the electronic density through oxygen from the carbonyl group, in addition to the nitrogen from the aromatic moiety, promoting closer hydrogen bonds. Also, methoxy groups have the ability to form hydrogen bonds.

Changing the methoxy group (**19**) at C6 to an amino group (**3**) leads to a decrease in IC_50_ values (0.39 and 6.2 µM respectively). In **3**, the presence of the amino group at C6 involves the same analysis in an internal hydrogen bond in compounds **20** and **21**, decreasing the DNA binding affinity value.

#### 3.1.1. Docking Studies

The telomerase holoenzyme is composed of three main units: a reverse transcriptase (telomerase reverse transcriptase, TERT), an RNA component (TR), and several species-specific proteins. The TERT protein is highly conserved across different species and shares a common domain organization, which can be divided into four consecutive structural domains: N-terminus domain (TEN), RNA-binding domain (TRBD), the reverse transcriptase domain (RT), and the C-terminal extension domain (CTE). TR varies considerably in size, sequence, and structure and contains (for human telomerase RNA) three main domains: the core domain (CR1–CR3), the CR4/CR5 domain, and the H/ACA scaRNA domain (CR6–CR8). The first two (core and CR4/CR5) bind the hTERT in TRBD independently. Methods applied to inhibit telomerase generally focus on targeting either TERT or TR [115].

We review some of the several studies on the structure of the units for *Tetrahymena thermophila* (tt), *Tribolium castaneum* (tc), or *Oryzias latipes* (ol) [25,26,116,117]. In the case of human telomerase, crystal structures of hTERT and hTR have not yet been reported; consequently, the molecular docking studies are based in non-humans [118,119]. The electron microscopy (EM) structure shows that the TERT unit allows an open and closed configuration for human telomerase, which in *Tribolium castaneum* is mediated by interactions between the RNA-binding domain (TRBD) and the thumb (two terminal domains of tcTERT), and is possibly required for telomerase ribonucleoprotein (RNP) assembly [25].

Docking studies to analyze the structure in tcTERT were conducted using molecular replacement with the substrate-free enzyme (PDB code: 3DU5 or 3DU6) [25]. These studies showed a conserved hydrophobic pocket or FVYL motif composed by Phe, Val, Tyr, and Leu residues (maintained invariant in hTERT). This pocket has an open configuration on the outer surface of the thumb domain, where part of the inhibitor (BIBR1532, naphthalene group) is located in a cavity between a part of Phe494, the backbone of Gly495, and alkyl side chain of Leu554. Van der Waals interactions are found with side chains of Met482 and Tyr551, along with a hydrophobic pincer form by the side chains of Phe494 and Ile550 that involves the other aromatic ring of the inhibitor (benzoic group). The aromatic groups in the inhibitor seems a crucial factor to consider.

In the case of TR, Chen et al. [67] analyzed the structure of the RNA component [26,116] with two oxoisoaporphine derivatives (**1** and **2**, Figure 1), using a low-resolution (6.5–8.0 Å) fluorescence resonance energy transfer (FRET) model of the hTR core domain (PDB code: 2INA) [26]. hTR is composed of four chains (a, b, c, and d) and the docking studies mainly showed interactions with chains a and d, where these chains helically wind and form a larger pocket. The oxoisoaporphine **1** has two hydrogen bonds with these chains; the first (at 2.0 Å) occurs between the nitrogen atom of the quinoline moiety and a hydrogen of the amino group of Cytidine151 and the second forms between the hydrogen of the hydroxyl group and an oxygen of the nucleoside moiety at 2.2 Å. Molecular coupling studies for compound **2** showed four hydrogen bonds, three formed by the nitrogen atoms in heterocyclic rings of **2** and Cytidine148 (2.1 Å), Cytidine149 (1.8 Å), and Adenosine150 (2.3 Å). The fourth hydrogen bond is formed by the oxygen atom in the carbonyl group and a hydrogen of the amino group of adenosine150 (2.2 Å). For these two additional hydrogen bonds, compound **2** is more embedded in this active pocket of chains a and d, showing lower binding energies (**1** = −6.43 and **2** = −9.76 kcal/mol). Besides the aromatic groups, this study showed the fundamental role of the hydrogen bond in stabilizing the complex oxoisoaporphine-RNA.

#### 3.1.2. Perspective

The oxoisoaporphine is a promising family of compounds in the development of new effective drugs against cancer. If we consider IC_50_ values of the oxisoaporphines derivates and we compare with other natural drugs such as resveratrol and curcumin [120,121], we observed interesting cytotoxic effect in common cancer line cell MCF-7 (breast cancer). These drugs exhibit values between 3 and 10 µM while the oxoisoaporphine derivates provide better anticancer activity with DNA interaction as the mechanism of action, at least one magnitude lower than commonly used natural products. The IC_50_ results (Table 1) show that the substituents influence the cytotoxicity of these compounds. The docking studies indicate weak interactions (H-bond or π-π stacking) to stabilize the TER-ligand complex and confirmed DNA-binding as the mechanism of action in this family of compounds. 

With the obtained results, certain concepts of the rational design of oxoisoaporphine derivatives can be inferred, which primarily include the substitution at C4, C5, and C6. The use of the electron donor groups in these positions increases the power of the aromaticity, favoring the π-π stacking interactions. The substitution at C9 could be considered, where the results show the use of a chain approximately five atoms long with a terminal group with the ability to generate H-bonds (NH_2_, OH, or SH). 

The combination of these structural modifications allows the generation of new compounds with a potential cytotoxic effect against tumoral cell lines.

### 3.2. Boldine and Derivatives

**Figure 6:**
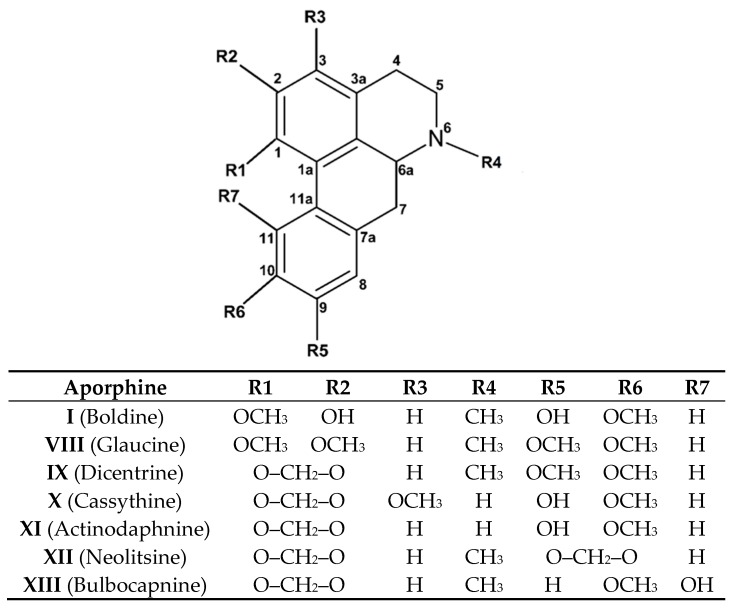
Derivatives molecules of boldine measured in cytotoxic and DNA interaction assays.

Natural derivatives of boldine, such as glaucine (**VIII**), dicentrine (**IX**), cassythine (**X**) actinodaphnine (**XI**), and neolitsine (**XII**) (Figure 6), have been studied due to the pharmacology and medicinal function cytotoxic action in several line cells, verified in vivo and in vitro for these known aporphine alkaloids [115,116]. Among the natural alkaloids, **I**, **VIII**, **IX**, **X**, and **XI** were studied by Hoet et al. [122], reporting an approach to structural–biological correlation of natural and commercial alkaloids.

The cytotoxicity evaluated on Hela cells evidenced the powerful action of **VIII** with IC_50_ = 8.2 μM, then **XI** and **X**, whose doses were two times more active, were followed by **IX** and **I** compounds. A reason for this finding could be that glaucine, with all peripheral methoxy groups, can stabilize the aromatic ring via resonance and favor interactions (binding or intercalation) through hydrogen bond interactions with some donor sites from DNA fragments. Simple UV absorption experiments hinted to the mechanism of action through binding interactions. For this series, bathochromic and hypochromic shifts in the absorption band at 305 nm were observed, indicating that **IX**, **X**, and **XI** have stronger interactions with DNA in this order: **XI**, **X**, and **IX** corresponding with IC_50_ increase value. This similar response can be attributed to structural considerations: For **IX**, **X**, and **XI**, the R1 and R2 positions are occupied by the methylenedioxy ring, whereas R6 is occupied by a –OCH_3_ group. However, the higher shift values on the absorption band agree with donor hydroxyl groups in R5 (compounds **X** and **XI**), which seem to favor DNA interaction. For more details such as shifts percentage, UV absorption experiments, please refer to Hoet et al.

Different hypsochromic shifts from **X** and **XI** may be due to the methoxy group in the R3 position stabilizing the aromatic ring via resonance. For **IX**, the response to DNA seems weaker, potentially due to the complete absence of a hydroxyl group in the peripheral positions, hindering hydrogen bonds with DNA fragments. Linear and circular dichroism experiments confirmed that this alkaloid with a methylenedioxy group in R1 and R2 positions has a lower reduced dichroism value compared with the value obtained for DNA alone (at 280 nm), reflecting the perpendicular orientation of the chromophore to the DNA helix axis. Thus, strategical substitutions with electron donor groups on the aromatic ring of the aporphine framework seem to favor intermolecular DNA interaction. 

Stevigny et al. evaluated the cytotoxicity of compounds **IX**, **X**, **XI**, and **XII** on another cancer line cell in vitro [123]. Specifically, when cytotoxic assay on the MeI-5 cell line was conducted, **X** showed the highest activity against cancer cells with an IC_50_ value of 24.3 μM and **XI** with 25.7 μM. On HL-60, cytotoxicity was achieved using 19.9 μM and 15.4 μM, respectively. Although the authors did not indicate any approach to determine a mechanism of action, they used camptothecine as a positive control, a known topoisomerase inhibitor.

The mentioned family of alkaloids differs in the conventional DNA intercalator structure, and often shows two or three fused aromatic rings that can confer rigidity. These alkaloids, including boldine, have two aromatic rings separated by a saturated ring, making them non-planar molecules. These aromatic rings can be substituted. Although these alkaloids are non-planar molecules, they intercalate weakly into DNA and may be adaptive intercalators, such as dicentrine **X** and boldine **I** derivatives, which undergo a conformational change to a planar conformation upon binding to DNA. 

#### 3.2.1. Exceptional Family of Compounds in DNA Presence

A molecular modelling study by Woo et al. revealed that **XI** has bent and twisted aromatic rings. As mentioned above, these two aromatic rings in some aporphines are surrounded by bulky groups such as methylenedioxy in R1 and R2 and hydroxy groups in different positions, including a saturated heterocyclic *N*-methyl group ring. This structure may hinder access of the molecule to intercalation sites. However, dicentrine, unlike the approach above, shows weak intercalation on DNA fragments according to the reduction value of the linear dichroism with respect to DNA alone. In the same sense, UV absorption measurements for bulbocapnine **IX** and **XI** compounds allowed the observation of a bathochromic shift upon the addition of DNA to each ligand solution, showing wavelength shifts to higher energy, with dicentrine having one of the higher values of the series [122]. These results, together with molecular modelling, demonstrated the ability of dicentrine to rotate around the single bonds at the 6a and 7 carbon atoms of the saturated rings, changing the bent structure to a flat conformation when in the presence of DNA. Even though boldine changes conformation and did not demonstrate DNA binding, its moderate response on DNA remains undetermined. 

Although boldine did not show significant DNA binding action, Gerhard et al. [7] evaluated the effect and underlying mechanisms of boldine on glioma proliferation and cell death in U138-MG and U87-MG human glioma cells. In this work, boldine was found to be an active cytotoxic agent when U138-MG cells were treated with increasing concentrations of boldine for 72 h, observing a decrease around 50%. Boldine arrests U138-MG cells in G2/M but does not cause apoptosis. However, cell cycle distribution analysis by flow cytometry allowed the observation that boldine increased the percentage of cells in the G2/M phase after 24 h. 

Noureini et al. [87] progressively evaluated the cytotoxicity and telomerase inhibitory effects of boldine and other derivatives in MDA-MB-231, MCF-7 cancer, and HEK293 non-cancerous immortal cell lines. In their work in 2015, they showed that boldine strongly suppresses proliferation in embryonic HEK293 kidney cells and two telomerase-positive breast cancer cells. The cytotoxic effect of boldine in human breast and kidney cells and telomerase positive immortal cells was measured in a dose- and time-dependent MTT assay. After 48 h, boldine showed a similar cell viability profile to berberine in an MCF-7 cell culture, a known telomerase inhibitor alkaloid. The cytotoxicity of boldine and berberine was determined with IC_50_ values of 160 and 54 μM, respectively, which demonstrates boldine antitelomerase efficiency to be at least comparable to that of berberine. Although the effects of boldine did not considerably change up to 60 μM, decreases were observed even using low concentrations at the same incubation time as berberine. The IC_50_ value for telomerase inhibition in human breast cancer and embryonic kidney cells was estimated (MDA-MB-231 at 29.1 μM and HEK293 and 38.8 μM) to be significantly lower than the 50% cytotoxic dose in an MTT assay (110 μM and 150 μM, respectively). Metastatic breast MBA-MB-231 cells were more sensitive at only 10 μM boldine with reduction in the telomerase activity at 39% than in other line cells (MCF-7 and HEK 293). In all cell lines, the IC_50_ for telomerase inhibition occurred when cytotoxicity was less than 10%.

When in vitro TRAP assays were measured, the reactions were treated with boldine in two different incubation times, 0 and 30 min before the correlated enzyme starts its activity, confirming inhibition by direct boldine–enzyme interaction, but this is not the mode of interaction.

In summary, boldine affects the regulation of mRNA (messenger RNA) at various points, although no interaction with telomere sequences has been detected for boldine. Therefore, the active telomerase content of the treated cells was reduced dose- and time-dependently through transcriptional downregulation.

**Figure 7:**
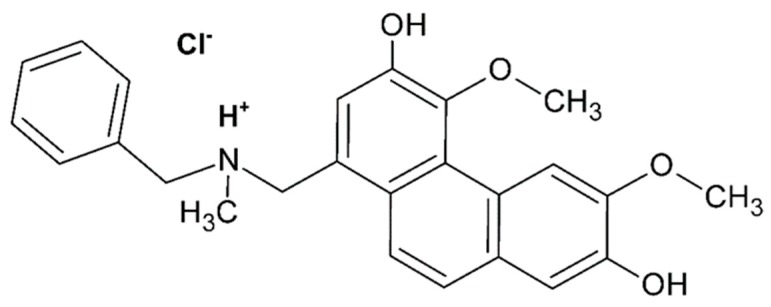
Chemical structures of *N*-benzylsecoboldine (BSB) hydrochloride [87].

Noureini et al. [87] analyzed the antiproliferative effects of boldine due to its potential in telomerase inhibition on several cancer cell lines and at non-toxic concentrations. They focused on where and how it may bind to the enzyme through docking by using a molecular dynamic simulation study of boldine and its derivative *N*-benzylsecoboldine hydrochloride (Figure 7).

For docking and molecular dynamic analysis, the crystal structure of TERT telomerase from tc was used because this enzyme has highly conserved motifs A and C in the reverse transcriptase palm region with amino acids in their sequences, especially in the active catalytic site. Firstly, the active sites were defined but the authors noticed that some extra binding site might exist. Therefore, focused and blind docking were used.

General results of telomerase inhibition by boldine and its derivative N-benzylsecoboldine showed that both ligands might bind to TERT in a binding box different from the active site, with inhibition constants of 9.15 µM and 0.221 µM, respectively. For boldine, the binding position reveals interactions with telomerase through three hydrogen bonds: between the NH group of Ala255 as donor and O^2^ of the boldine framework as acceptor, H atom of asparagine Asn369 as donor and O^4^ (from methoxy group) of the ligand, and the H atom in the amide group of Lys372 as a donor atom to a hydroxyl oxygen atom of the ligand. For N-benzylsecoboldine, BSB binds to TERT via one hydrogen bond between O1 (methoxy group) of the ligand and the NH hydrogen atom of Ile252 of telomerase at 1.4 Å.

#### 3.2.2. Perspective

Data obtained from computational experimental studies, and various reports of the structure–activity support the inhibitory potencies of boldine and its derivatives, especially in those structures that present hydroxyl y/o methoxy groups in the periphery of aporphine due to the ability to sense metabolic environment in reductive conditions. 

On this matter, some common natural drugs could be compared with the anticancer effect of boldine derivates. Cassytine and actinodaphtine (**X**, **XI**) showed IC_50_ values at similar doses to resveratrol, butein, simvastatin, and thymoquinone in HL-60 cell lines [124,125,126] although these differ in their mechanisms of action, such as the activation of caspase-3 or generation of ROS. For this reason, the research remains continuously active, mainly due to the synergistic mechanism of replication in the uncontrolled proliferation of cancer cells, posing a considerable challenge to current analysis tools. 

### 3.3. Sampangine: Anticancer Activity and Action Mechanism

Bailly et al. were the first to evaluate the antitumoral activity of sampangine. They assessed the effect of sampangine on the cell proliferation and viability of HL-60 leukaemia cells [127]. In this study, sampangine showed activity against HL-60 cancer cells with an IC_50_ value of 2.65 μM and 24.47 μM cell proliferation and viability, respectively. The ratio between both values was less than one, indicating that apoptosis is the type of cell death induced by the sampangine treatment. To confirm the induced apoptosis, they monitored the effect induced by sampangine on the cell cycle. The HL-60 cells were treated for 48 h with different concentrations of sampangine. The cells that were treated with 40 μM of compound showed an accumulation of cells in the G0/G1 phase from 40% in the control up to 69% in the drug-treated samples. They observed that the S-phase cell population decreased from 49% to 18%. The cells treated with 20 μM sampangine showed a hypodiploid DNA content peak of 75% sub-G1. This cell population is characteristic of the loss of DNA content that is associated with apoptosis as a mechanism of cell death. To complement these results, they measured the effect of sampangine on the activity of caspase-3, a key enzyme in the apoptosis process. The assays showed that cancer cells treated with 20 μM sampangine displayed a massive activation of caspase-3, a result that is consistent with the induction of apoptosis. Based on the hypothesis that apoptotic stimuli alter the mitochondrial transmembrane potential (ΔΨ_m_), they monitored the changes in ΔΨ_m_ induced by sampangine. After 48 h of incubation, they observed a marked hyperpolarisation of the mitochondrial membrane when the HL-60 cells were treated with 4 μM of sampangine, and depolarization with 20 μM of the compound. From these results, ROS, probably hydroxyl radicals, may be contributed from the accumulation of intracellular hydrogen peroxide correlated with the abnormal activity of the mitochondrial respiration chain [128].

The hypothesis of the production of oxygen-based free radicals by sampangine was based on the results previously reported for ascidemin. Ascidemin is a structural analogue of sampangine and has be shown to be highly cytotoxic to several types of tumour cells, exhibiting potent pro-apoptotic activities [129]. The mechanism of action is associated with the production of ROS responsible for direct DNA damage [130]. The authors studied the ability of sampangine to generate ROS in HL-60 cells to relate this with apoptosis induction through mitochondrial perturbation, caspase activation, and nuclear degradation. The HL-60 cells were treated with a concentration of 1 to 20 μM sampangine for 48 h; the activation of caspase-3 was directly proportional to the drug concentration. With 20 μM sampangine treatment, 56% of the cell population participated in the caspase-3 activation. Also, they found a direct correlation between caspase activation and apoptotic nuclear alteration when observing enzyme activation and the appearance of sub-G1 cells in the cell cycle measurements simultaneously. They observed that both effects started to appear at 4 μM and that the subG1 alterations induced by 20 μM were fully inhibited by Z-VAD (inhibitor of caspase activity). This result confirms the induction of apoptosis by sampangine.

The relationship between sampangine and apoptosis is based on the oxidant stress hypothesis due to the presence an iminoquinone moiety in sampangine, which has been implicated in drug-induced oxidant stress activation in cells as reported in ascidemin [130]. To evaluate the redox state, the production of ROS was measured using hydroethidine, whose generation in HL-60 cells was dose-dependent. The oxidative stress started at 4 μM sampangine (in 23% of treated cells) reaching 69% with 20 μM. To confirm the implication of ROS in these experiments, different antioxidant molecules were included, and they observed that the oxidative burst induced at 4 or 20 μM sampangine fully decreased in the presence of *N*-acetyl cysteine and partially with vitamin C or vitamin E. The ROS generation induced by sampangine in Z-VAD-treated cells was also measured, which did not modify ROS production, suggesting that the caspase activation previously observed was not involved in oxidative stress. However, the results obtained from these experiments indicated that ROS play an important role in sampangine-mediated apoptosis. The authors established a method to relate the time of the ROS production to the sampangine concentration. They conducted various time-dependent experiments comparing sampangine behavior with H_2_O_2_ (a potent oxidative agent). A total of 91% of the HL-60 cells incubated with 20 μM sampangine produced ROS after 30 min, which decreased quickly for the first four hours and remained constant around 60% until 48 h. With a higher sampangine concentration (40 μM), ROS could be induced after two hours of treatment of HL-60 cells.

Identical to Matsumoto et al., Bailly et al. evaluated mitochondrial alterations during sampangine treatment (ΔΨ_m_) but using a JC-1 probe [99], considered as one of the most mitochondria-specific probes [131]. The cells treated with 20 μM sampangine for 48 h showed a massive population of cells with depolarized mitochondria (92%). To establish a link between the mitochondrial events and oxidative stress, the inhibition of mitochondrial alteration was examined using cyclosporine A, an inhibitor of the permeability transition that prevents the apoptosis-associated decreased in ΔΨ_m_ [132]. The results obtained suggested that the mitochondrial alterations previously observed are not associated with a permeability transition. However, using the protonophore ClCCP (uncoupling agent that inhibits mitochondrial alteration in nanomolar concentration), ROS production was found to not originate from mitochondria.

To test this hypothesis, Nagle et al. performed essays to determine the effects of the sampangine on cellular respiration in *S. cerevisiae* (budding yeast) and human breast tumor T47D cells [100]. The genome-wide screening of yeast deletion mutant libraries revealed that the observed hypersensitivity is associated with mutants that affect mitochondrial function, in particular, the ATP synthase [133]. The oxygen consumption rate was measured in the presence of sampangine for both cell systems, and the results showed that sampangine stimulates cellular oxygen consumption in a concentration-dependent manner in tumor cells and yeast *S. cerevisiae*. To determine if sampangine stimulates respiration via protonophore-based uncoupling or via stimulating cellular ATP consumption, the effect of sampangine on oligomycin-induced state 4 respiration in T47D cells was studied. Oligomycin decelerates mitochondrial electron transport, which is reflected by a decrease in the rate of oxygen consumption, by increasing the proton gradient across the inner mitochondrial membrane. Studies showed that sampangine overcame oligomycin-stalled cellular oxygen consumption in T47D cells, which is consistent with stimulating ATP turnover. To further test the possibility that sampangine uncouples, the authors monitored the mitochondrial membrane potential using fluorescent lipophilic cationic dye TMRM^+^. Mitochondrial TMRM^+^ accumulation was modestly affected by sampangine, suggesting that this compound may only weakly uncouple. Sampangine increasing oxygen consumption with minimal perturbation of mitochondrial TMRM accumulation suggests that sampangine may primarily increase non-mitochondrial oxygen consumption. Thus, the authors studied the effect of sampangine on cellular oxygen consumption in the presence of sodium azide, an electron transport chain complex IV inhibitor that suppresses mitochondrial oxygen consumption. However, sampangine stimulated sodium-azide-incentive oxygen consumption to nearly the same extent as without azide, suggesting that much of the increase did not depend on complex IV and is therefore non-mitochondrial.

Often compounds that include quinone and anthraquinone groups in their structure produce ROS through a redox cycling process that can be enhanced by coupling the reaction with ascorbate oxidation/reduction [134,135,136,137,138]. Therefore, the effect of ascorbate on sampangine-induced oxygen consumption using the T47D cell-based respiration essay was examined. The results supported that sampangine stimulates non-mitochondrial oxygen consumption and ascorbate facilitates the quinone/anthraquinone-like redox cycling of sampangine. 

The hypoxia-inducible factors (HIFs) are the principal regulators of oxygen homeostasis and induce cellular responses to decreased oxygen tension at the transcription level [139,140]. In particular, HIF-1 is activated by hypoxic conditions and the mitochondrion constitute an important component in the signalling network that regulates their activity [141,142,143,144]. The effect of sampangine on HIF-1 activity in T47D cells was evaluated following the results reported by Du et al. [144]. Sampangine enhanced the hypoxic activation of HIF-1 at lower concentrations and inhibited it at higher concentrations. Mitochondria generated ROS are known to mediate hypoxic HIF-1 activation, and possibly sampangine stimulated ROS production and enhanced hypoxic signalling at a lower concentration. This cytostatic/cytotoxic effect was confirmed in a standard 48 h of exposure to sampangine on estrogen-dependent T47D cells.

**Figure 8:**
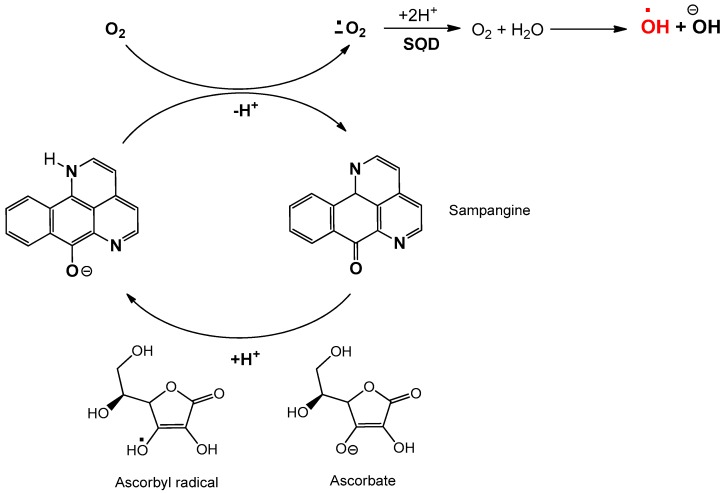
Once cycle in ascorbate-coupled redox cycling of sampangine to produce reactive oxygen species (ROS) [99].

In summary, sampangine increases cellular oxygen consumption through a non-mitochondrial process that likely involves redox cycling of this compound. As sampangine is structurally analogous to the electron carrier ubiquinone, this compound could be reduced to a reactive semi-iminoquinone form, e.g., by complex I, that then readily reduces oxygen to superoxide. As shown in Figure 8, ascorbate could be an electron donor for redox cycling and ROS generation [134,135,136,137,138]. These results agree with a previous observation by Baille et al. that sampangine burst apoptosis in HL-60 cells is ROS-associated [99].

Recent attempts to improve the therapeutic potential of sampangine via structural modification have produced thiophonequinones that retain much of the antifungal and cytotoxicity activity, but no longer require the complexity an iminoquinone heterocycle such as ascididemnins [130,145]. Sampangine-induced redox cycling and its associated ROS-induced oxidative stress may occur with cellular signalling pathways such as those that regulate HIF-1. 

Agarwal et al. and Huang et al. highlighted that the inhibition of heme biosynthesis could be the principal mechanism of action of sampangine [133,146]. Heme is present in complexes **III** and **IV** of the electron transport chain and possibly affects the heme dysfunction in sampangine-induced ROS production, as iron and various heme biosynthetic intermediates are known to be oxidized via reaction with cellular ROS [147,148,149,150].

Nasiri et al. demonstrated that sampangine produces ROS in vitro without the involvement of cellular components in a heme-independent manner. They evaluated the ROS production using DTT (dithiotheitol) as a mild reducing agent and observed that radical production occurs directly on the iminoquinone toxicophore of sampangine [32]. The planar geometry of sampangine suggests the potential DNA interaction through intercalation between adjacent base pairs. Peterson et al. evaluated this interaction and observed a relative weak binding affinity (K_b_ = 3 × 10^3^ M), although these results were never published [151]. Based on this approach, Naisiri et al. evaluated the interaction between sampangine and the biomolecule [32] to confirm the low interaction affinity, suggesting that DNA is not a potential target of sampangine.

#### 3.3.1. Sampangine Derivatives

As mentioned above, sampangine was isolated in 1986 from the chloroform extract of the stem bark of *Cananga odorata,* which was obtained in a 0.001% yield. Three years later, Breacher reported the total synthesis of sampangine via the reaction of a cleistopholine derivative with ammonium chloride in glacial acetic acid [152]. The experimental process produced pure sampangine with a 26% yield and this marked a significant breakthrough in sampangine chemistry. The first studies of the biological properties of sampangine were conducted by Peterson et al. in 1992 [153]. They evaluated antifungal activity against *Candida albicans* and *neoformans* obtaining MIC values of 1.56 and 0.78 μg/mL, respectively. Although sampangine was later inactive in the in vivo evaluation in a mouse model of cryptococosis, the in vitro results provided an approach for the development new sampangine derivates with new and better biological properties.

**Figure 9:**
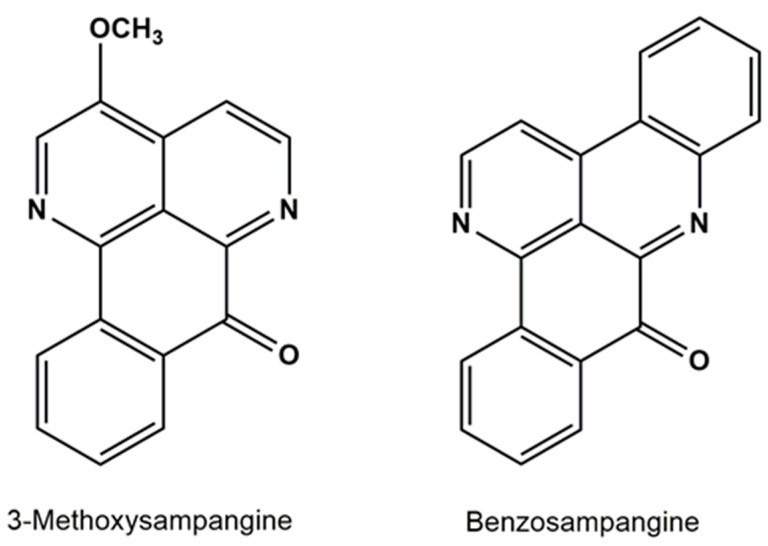
Chemical structure of 3-methoxysampangine and benzosampangine.

Sampangine derivates have been synthetized as potential antifungal (or antimycobacterial) agents. Orabi reviewed more than 30 derivatives of sampangine and their antifungal activities. Orabi also developed the metabolic profile of sampangine, 3-methoxisampangine, and benzosampangine, which were mentioned as important analogues of sampangine (Figure 9) [154]. 

In 1990, Liu et al. reported the isolation of 3-methoxysampangine from the root bark the African west tree (*Cleistopholis patensk*) and elucidated the structure and in vitro antifungal activity [155]. The isolation of 3-methoxysampangine was obtained in a 0.000156% yield. Therefore, many attempts were made to synthesize it with a better yield. As with sampangine, Peterson et al. reported the total synthesis (and the evaluation of the antimycotic and antimycobacterial activity) of 3-methoxysampangine with a 0.17% yield. However, in 1997, Zjawiony et al. produced this compound with a 40% yield using another procedure [156]. Benzosampangine has not been reported to occur in nature; however, it was also synthesized by Peterson et al. in 1992. This compound showed an important antifungal activity in vitro (MIC = 0.39 μg/mL and 1.56 μg/mL against *C. albicans* and *C. neoformans*, respectively), and in vivo evaluation showed that benzosampangine is capable of significantly reducing the brain tissue of *C. neoformans*.

**Figure 10:**
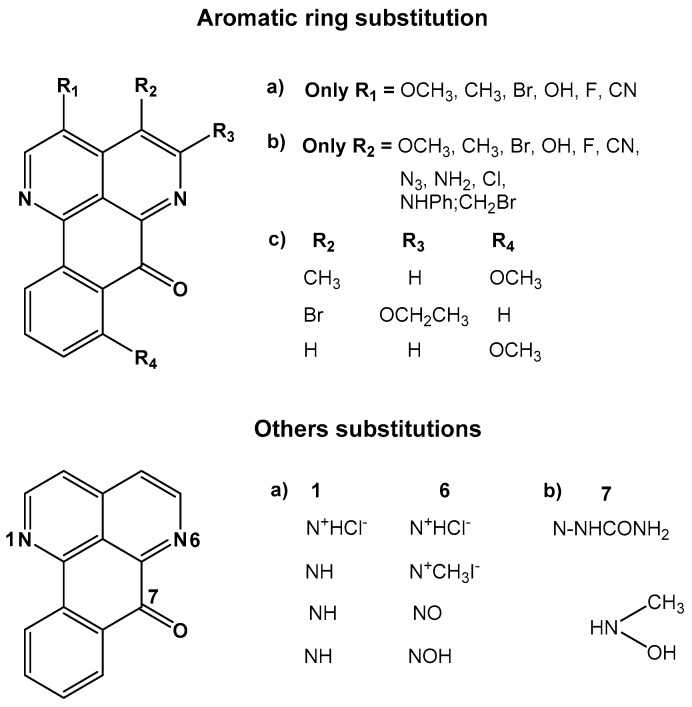
Chemical structure of sampangine derivatives [154].

The derivates of sampangine are categorized according to the position of substituents in the structure. The chemical structures of these analogues are shown in Figure 10.

In general, sampangine derivatives have been produced through nucleophilic and electrophilic substitution reactions [101,153,156,157,158,159,160]. The analogues that have been synthesized correspond to those that have only one substituent in the basic structure of sampangine, particularly in the A or B rings (see Figure 4). Substitution at C3 produces six different analogues and the substitution at C4 generates 12 new compounds. Including two or more substituent in both the A and B rings is possible, which produces three other derivates of sampangine. Other derivates of sampangine have been synthesized by including a substituent in other positions of the structure, specifically at N6 and C7. These substitutions change the N6 reactivity and allow replacing the quinone group of the sampangine structure with another functional group, such as oxime and semicarbazone, among others.

The antifungal and antimycobacterial activities of these sampangine derivates have been evaluated and summarized in Orabi’s review. From the in vitro evaluation of the antifungal activity of these analogues, a structure–activity relationship can be found. The biological activity is affected by the position and type of substituent in the sampangine structure, producing MIC values between 0.1 and 100 μg/mL. The modification of the quinone group generates a decrease (even total loss) in the activity. However, sampangine-7-oxime and 7-semicarbazone showed good activity related to the microbial and mammalian metabolites of sampangine (as mentioned in these reviews). The antifungal activity depends on the yeast type used in the evaluation. The derivate substituted at C4 reduces the anticandidal activity, whereas some 4-substituted analogues retain the activity against *C. neoformans*.

**Figure 11:**
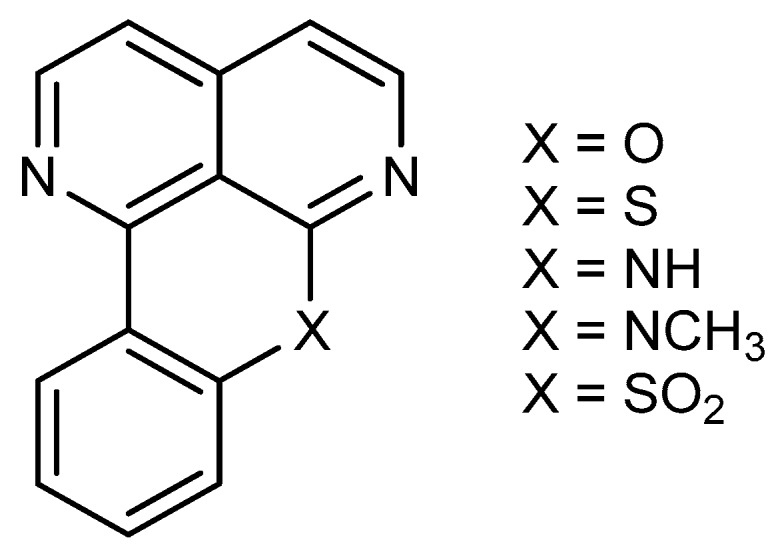
Sampangine derivates by modification of the C7 group [161].

Mink and Bracher synthetized five new derivates of sampangine by modifying only the quinone group in the structure of sampangine (Figure 11). The in vitro antifungal evaluation showed that sampangine has activity comparable to clotrimazole (used as an antimycotic drug reference), in that the oxo derivate and thio compound were almost inactive, whereas both amino analogues and sulfone showed high and broad spectrum activities compared with sampangine and clotrimazole [161]. 

Following Orabi’s summary, Claes et al. synthesized six new analogues of sampangine by substitution of different functional groups at the C1, C4, and C5 positions (and retaining the quinone group). They evaluated the potential activity against different *Mycobacterium* systems [159]. The in vitro evaluation showed that, in general, the analogues have less activity than sampangine, but they did not explore the structure–activity relationship to explain this behavior. 

Jiang et al. synthesized a series of D-ring scaffold hopping derivates of sampangine including different heterocycles, such as furan, thiophene, and pyrrole, to replace the phenyl group (D-ring). They focused on improving the antifungal activity and water solubility of the compounds of the sampangine and to obtain data for SAR studies (structure–activity-relationship). The in vitro antifungal assays revealed that most of the compounds showed broad-spectrum inhibitory activity against the studied human fungal pathogens. The thiophene derivate was more active against all fungi systems evaluated, showing potent fungistatic activity with a broad spectrum (MIC = 0.25 to 8 μg/mL) in contrast with other furan and pyrrole analogues, which showed a decrease in the antifungal activity. The compounds showed better solubility in water than sampangine, and the most active compound also exhibited the best water solubility (48 μg/mL) [118].

Currently, studies on the anticancer activity of sampangine derivates are lacking. However, Nasiri et al. related the ROS synthesizing derivates and evaluated the ability to produce ROS in vitro as part of their potential mechanism of action against cancer cells [32]. The compounds included partial 1,4- and 1,2-iminoquinone structures, dimethoxy derivates, and an analogue to an extended aromatic system. These studies identified the 1,4-iminoquinone scaffold as responsible for ROS production, similar to the behavior of ascidemin previously mentioned.

#### 3.3.2. Perspective

In summary, from the results of the structure–antifungal activity relationship of the sampangine derivates and ROS production ability, this behavior of sampangine could be indicative of anticancer activity. As has been observed, the modification of the quinone group generates a decrease in antifungal activity, except for the compounds that include a substituent group that can produce ROS in biologic media, such as semicarbazone. The inclusion of a thiophene group to replace the D-ring increases water solubility and could have a positive effect on the anticancer activity. Therefore, in the design of new sampangine derivates with potential anticancer activity, we recommended retaining the quinone group because is an important factor in the antitumoral activity of sampangine and including substituents that increase water solubility. Concerning the clinical advances of sampangine and the studies that illustrate the regulatory pathways involved in the mechanism of action of sampangine (ROS production), side effects and toxicity may significantly limit the clinical potential of sampangine and its analogues as antifungal and antitumor agents.

Although more extensive studies are needed, we believe it is possible to place sampangine and their derivates as an interesting mechanism for anticancer research if we considered commonly used natural drugs such as simvastatin, celastrol, and reverastrol [120,124,162], whose cytotoxicity values are similar in leukemia cell line HL-60. 

## 4. Final Comments

The oxoisoaporphines and their derivatives stand out for their cytotoxic versatility in several cell lines, standing out in the breast cancer cell line even against natural drugs of common use.

Boldine and derivatives, on the other hand, although not as versatile as the oxoisoaporphine family, maintain attention due to their natural and endemic origin and their good performance against the interaction of DNA due to their plasticity.

Although Sampangine’s anticancer function has been measured on leukemia cell lines only, it has a good cytotoxic response at a similar dose to some anticancer natural drugs. Moreover, sampangine stands out among the oxoisoaporphine and boldine derivates, thus shifting attention towards additional assays on other cancer cell lines and pharmacokinetic studies for this family of compounds.

## Figures and Tables

**Table 1:** Toxicity test of oxoisoaporphine derivatives in several line cancer cells.

Cell Line	IC_50_ (μM)																							
	1	2	3	4	5	6	7	8	9	10	11	12	13	14	15	16	17	18	19	20	21	22	23	24
T-24	38.4	35			16.8																			
Hep-G2	20.5	18			17.4	19.7	8.14	9.7	6.4	12.1	21.02	3.9	8.2	7.7	12.9	29.8				128.5	141.2	108.3	60.6	127.3
SK-OV-3	>100	40			>100	130														141.9	143.6	154.1	55.9	90.2
SK-OV-3/DDP	13.9	17			36.8	85																		145.1
BEL-7402	54.7	50			36.7	95																		69.0
NCI-H460	66.9	57			>100	35.5	2.1	9.2	4.2	5.0	30.3	2.4	16.7	6.7	9.1	19.3				95.3	94.7	91.0	88.1	72.4
HCT-8	60.2	56			>100	80														112.5	135.8	167.1	42.7	52.3
HL-7702	64.5	75			>100	100														98.1	101.2	100.1	132.2	99.7
A549			>50	8.8																108.4	86.1	115.2	72.2	
HL-60			>50	>50																				
MCF-7			6.2	3.0		20.4	11.8	17.5	5.1	12.7	28.1	4.3	8.0	9.1	27.4	11.5	33.1	>100	0.39					122.9
P-388			9.6	30.5																				
H460																	45.2	>100	3.18					
HT-29																	>100	>100	>100					
